# From ontogenesis to clinical practice: waking up to infant sleep

**DOI:** 10.1093/sleepadvances/zpad017

**Published:** 2023-04-11

**Authors:** Thomas F Anders

**Affiliations:** Distinguished Professor Emeritus, University of California, Davis CA, USA; Adjunct Professor of Psychiatry and Behavioral Sciences, Stanford University School of Medicine, Stanford CA, USA; Adjunct Professor of Psychiatry and Human Development, Warren Alpert Medical School, Brown University, Providence RI, USA

**Keywords:** videosomnography, infant sleep, ontogenesis, infant/toddler sleep problems, intervention

## Abstract

This article describes the author’s research journey exploring infant and toddler sleep. From polygraphic recording in hospital nurseries to using videosomnography in homes, the author traced the longitudinal development of infant/toddler nighttime sleep and waking behaviors. The home-based video observations led to a redefinition of a pediatric milestone; namely, “sleeping through the night,” and provided a framework for assessing and treating infant/toddler nighttime sleep problems.

**Statement of Significance**

This is a description of one investigator’s pathway through several decades of sleep research—how he and the field both evolved, and the particular fascination of unexpected observations.

## Introduction

In July of 1959, the Stanford Medical School was in San Francisco and I was starting my 3rd year. My first rotation that July was a 6-week clerkship in Internal Medicine. As a very nervous new student doctor, I met my intern, a recent Columbia University graduate, Howard Roffwarg (whom everyone called Howie). He was also newly minted and also nervous, an East coast transplant to the Wild West. Needless to say, we struck it off and he made my first clerkship very enjoyable and meaningful. As a native San Franciscan, my tips for fun in the Bay Area made his year in the city enjoyable as well.

Fast forward to 1963, I was stationed in Tripoli Libya as a US Air Force flight surgeon and pediatrician after having been drafted during the middle of my pediatric residency at Boston Children’s Hospital. While in Libya, I decided to switch my focus and pursue a career in psychiatry rather than return to complete my training in pediatrics. What a pleasant and serendipitous surprise it was to find that my interviewer for a Columbia psychiatry residency position was my old friend Howard Roffwarg. I joined the Columbia program as a first-year resident in July 1964. With a budding psychiatrist’s interest in dreams, a pediatrician’s interest in development, the neurobiological excitement of the discovery of Rapid Eye Movement (REM) (dreaming) sleep and a friend and mentor who was a leading investigator in sleep state ontogenesis, I found myself in a perfect storm. My path to infant sleep research was set.

## Polygraphic Recording of Infant Sleep

From 1964 to 1967, as a resident psychiatrist at the New York State Psychiatric Institute, I spent several nights a week, as a researcher, polygraphically recording the sleep of newborn infants in the Columbia Babies Hospital nursery. We were attempting to expand the data set that Howie, with Joseph Muzio and Bill Dement, had so elegantly reported on in their seminal ontogenesis paper in *Science* [1]. When Howie, moved to join the Department of Psychiatry at Montefiore Hospital/Albert Einstein School of Medicine (AECOM), I followed him (1967–1972), first as a NIMH T-32 supported research fellow then as an Assistant Professor.

During those early years, I found myself among a small but collegial handful of worldwide infant sleep researchers (Robert Emde, Arthur Parmelee, Evelyn Thoman, Lou Sander in the United States, Klaus Minde in Canada, Olga Petre-Quadens in Belgium, Colette Dreyfus-Brisac, Nicole Monod and Lucille Garma in France, Heinz Prechtl in Holland and Jaroslava Dittrichova and Hanus and Mathlilde Papousek in Czechoslovakia). One of my first endeavors was to develop an infant sleep-scoring manual modeled after the classic Rechtschaffen and Kales manual for scoring adult sleep [2]. I was hopeful that with infant sleep studies beginning to emerge around the world, development of a standardized scoring system would be useful. Over several years and numerous meetings and get-togethers at APSS and elsewhere, I published, with Drs. Emde and Parmelee, the Infant Sleep Scoring Manual which defined and rendered pictorially the polygraphic characteristics of Waking, Active Sleep (AS), Quiet Sleep, and an immature “state” that we called Indeterminate Sleep [3]. Our presumption was that the three developing sleep states would, over the first 6 months of life, mature into REM sleep and the four Non Rapid Eye Movement (NREM) sleep stages typical of adult sleep.

During the early 1960s, mainframe computing was also appearing in academic medical centers. Working with one of the computer scientists, another early objective of mine was to automate the process of portraying sleep–wake histograms. After manually scoring each 30-second epoch of polygraph recording, then punching each epoch onto an individual IBM card and finally loading a huge stack of these cards into a main frame card reader, RIPVAN, our computer program would print a sleep histogram and compute the relevant metrics [4]. With the subsequent rapid advances in computer technology, however, my early forays into computer automation quickly became obsolete. My interest in automated technologies, nevertheless, persisted.

While at Montefiore, I was able to obtain project funding from the W.T. Grant Foundation and a Career Development Award from the National Institute of Mental Health (NIMH). My lab moved from the newborn nursery at Morrisania Hospital to the newly constructed Rose F. Kennedy Research Center on the campus of the Albert Einstein College of Medicine (AECOM). An eighth-floor bridge connected my lab at the R.F.K. Center to the newborn nursery and my polygraphic studies continued.

Since mothers were reluctant to bring their newborns back to a sleep lab once discharged home, my studies were confined to short-term protocols for the 2–3 days that an infant remained in the newborn nursery. We published reports on brief AS deprivation and brief total sleep deprivation demonstrating that newborns exhibited REM rebound as well as preferential NREM rebound after total sleep deprivation [5]. We also reported that newborns had a robust cortisol response to a painful stimulus such as circumcision and that they had a growth hormone response during sleep [6–8].

The major frustration for me at AECOM was the difficulty in mounting longitudinal studies. At that time, polygraphy in a sleep laboratory was the only way of studying sleep, and parents of typically developing young infants, as mentioned above, were not eager to return for further recordings. My primary interest, however, was to explore, longitudinally, whether the trajectory of REM and NREM sleep development over the first few years of life might predict or be associated with neurodevelopmental outcomes, and/or whether young participants with suspected atypical neurodevelopment might demonstrate markers in their sleep–wake state organization [9].

A non-sleep-related blessing of working with Howie at AECOM was meeting my future wife in his lab. Constance (Connie) Bowe was working in Howie’s animal lab at Montefiore Hospital, studying the ontogenesis of REM sleep in kittens. We married in 1970 and in 1972 we moved to the State University of New York at Buffalo where she entered the first-year medical school class and I opened my sleep lab at the Buffalo Children’s Hospital.

## Video Recording of Infant Sleep

As luck would have it, our first child was born soon after arriving in Buffalo; my first projected participant for a longitudinal study! Alas, after facing the dilemma of placing electrodes on my own newborn, I searched for a less invasive way to study infant sleep. With the help of Professor Arnold Sameroff of the University of Rochester and his graduate student Anita Sostek, we introduced time-lapse video recording to the study of infant sleep. Time-lapse video recording had just come onto the popular market and allowed us to continuously record an entire night’s sleep on a single video cassette. This methodology also allowed us to record in the home without instrumenting the infant and to return repeatedly to the home for longitudinal studies.

Having sat and watched a large number of babies sleeping while being polygraphically recorded over the preceding years, I was confident that the Waking, AS (REM), and quiet sleep (NREM) states could be coded from behavioral observations of the videotapes. The first challenge was to develop coding rules for the video somnoograms. We coded small and large body movements and observed rapid eye movements, periods of prolonged, sustained open eyes, and crying vocalizations. Using a 5-minute sliding window or smoothing interval to determine state changes, we were able to define waking and sleeping states. Before beginning our home studies, still in the newborn nursery, we used polygraphy and time-lapse videosomnography simultaneously in order to establish adequate reliability and validity of our video behavioral coding [10, 11]. Our scoring rules for the videotapes were significantly and meaningfully correlated with our scoring of the polysomnograms.

The video system consisted of a rather heavy and cumbersome commercial time-lapse video cassette recorder, a video camera on a tripod, and an infra-red-light source, all of which we carted from the lab to families’ homes. We instructed the parent on how to activate the recorder and for multi-night recordings, we provided extra video cassettes, one for each night. Parents also completed a sleep diary. We remained in contact with the family on a daily basis and were always available to troubleshoot problems which, in fact, were minimal.

There were some obvious downsides to the video methodology. The video recorder was bulky and heavy and difficult to transport to homes from the lab. The camera recorded only sleep when the baby was in the crib and, thus, during times when the baby had been removed for a period, an empty crib was recorded. Also, video recording required that the babies sleep mostly uncovered or lightly covered and alone. Co-sleeping families were not included in our studies. Since naps were routinely not recorded, the relationship between daytime and nighttime sleep was not investigated; an area that still requires further study. In addition, our studies were mostly carried out with educated, two-parent, middle-class families, so were representative of only a small sector of the population; albeit a population that brought their infants to the clinic for disrupted sleep. Finally, scoring the tapes back in the lab was tedious. Initial scoring often took 3–4 hours per recording night. After training our research assistants to reliability on several standard tapes, we always had each night scored by one research assistant with a second research assistant (RA) re-scoring 20% of that night. I triple-checked many of the tapes.

After two 2 years in Buffalo establishing the video technology and insuring its feasibility, reliability, and validity, our lab moved to Stanford in 1974 where our home-recorded, longitudinal studies began in earnest. These studies recruited infants in the nursery and then followed them at regular intervals through the first 12 months of age. At each time point, we obtained three consecutive nights of sleep. We also recruited a cohort of preterm infants around 36 weeks of conceptional age, and recorded them at the same time points corrected for gestational age. In general, our results supported the findings of other polygraphic reports that the proportion of REM sleep decreased during the first-year of life as NREM sleep increased proportionally. The REM sleep onsets that were characteristic of newborn sleep were replaced by NREM sleep onsets around 3 months of age. Also, in these studies, we found no discernable differences in these ontogenetic changes between full-term and preterm infants [12–15]. At Stanford, the collegiality and support of our work from Bill Dement, Christian Guilleminault, and Mary Carskadon were greatly appreciated.

## Wakeful Babies; Sleeping Through The Night

During the Stanford years, the “sleep problems” of infants and young children began to populate the news media and plague pediatric practices. Night waking during the first years of life and delays in “sleeping through the night” were reported by families as disruptive to family life. Reports in the scientific literature suggested that 30%–50% of infants presented with these night waking problems during their first years. Moreover, at this time, pediatric sleep researchers were beginning to develop a nosology of sleep disorders for children: the behavioral insomnias, parasomnias, and REM sleep disorders. Nightmares were distinguished from Night Terrors and Somnambulism was distinguished from a Functional Disorder. Narcolepsy was defined.

Serendipitously, our time-lapse videosomnography provided us with two significant insights into these clinical issues. The videos provided us with a look at the parent-infant interactions around bedtime and falling asleep; and, the videos provided us with views of the awake baby in the crib, either crying or lying quietly awake in the middle of the night. Frequently an awake baby lying quietly returned to sleep after 3 to 5 minutes independently without the knowledge of her parents; whereas the crying baby was able to elicit parental interventions. Although we obtained parent-completed sleep diaries for each night, their records were often not accurate. Especially for older infants, we found that what a parent reported about her sleeping infant was often not what the camera saw.

As our interest shifted more to falling asleep at the beginning of the night (sleep onset) and night waking after sleep onset, the scoring of video tapes shifted to scoring only sleep and waking states rather than REM and NREM states. We also began scoring the parent-infant interactions at the beginning of the night and in response to the middle-of-the-night awakenings. This shift to the contextual and interactional aspects of infant sleep was strengthened as a result of my spending the 1984–1985 academic year as a Fellow at the Center for Advanced Studies in Behavioral Sciences (CABS) at Stanford. With colleagues Robert Emde, Arnold Sameroff, Alan Sroufe and Arthur Parmelee and consultants Daniel Stern and David Reiss, I learned a lot about relationships; their importance for development, how they expressed themselves and how disturbances could manifest in the context of the infant’s interactions and environment [16]. Attachment theory as promoted by John Bowlby and Mary Ainsworth, among others, also permeated our work that year at the Center [17, 18].

While at Stanford, although my interest in infant/toddler sleep never waned and my lab remained active thanks to a number of graduate students, my attention and, to some extent, my identity shifted to that of a clinical, academic, child psychiatric administrator. As Child and Adolescent Psychiatry Division Chief, my responsibilities and commitments broadened significantly. Also, three young sons and my wife, Connie’s medical career filled the days and nights. When Connie finished her pediatric and neurology residencies, we moved again.

In 1985, the family moved to Providence RI where my video-sleep lab was set up at Bradley Hospital, a child and adolescent psychiatric facility affiliated with Brown University. I was, indeed, fortunate that my colleague from Stanford, Mary Carskadon, PhD, joined me and set up her polysomnography lab as a part of the Bradley/Brown partnership.

## Signaling and Self-Soothing Awakenings

The home-based, time-lapse video recording studies continued but with a new longitudinal focus. I was interested in how and when babies learned to “sleep through the night,” as defined by their parents, and how parent behaviors at bedtime and their responses to middle-of-the-night awakenings might relate to their infants’ sleep outcomes. In other words, how did the parent-infant relationship influence the transition from fragmented to consolidated sleep for the infant, and what factors were associated with the frequently reported, common “sleep problems” of infancy? Textbooks of Pediatrics, at the time, reported that typically developing infants should “sleep through the night” by 6 months of age.

Our video recordings taught us much. First, we learned that throughout the first-year of life, very few babies truly “sleep” through the night. By 6 months of age, the longest sustained sleep period (LSP) on average had lengthened from 3 to 4 hours at birth (the inter-feeding period) to 6 hours and the LSP did not lengthen further during the second 6 months of life. Instead, we noted that the nature of the awakenings changed. At the youngest ages, most of the awakenings were associated with crying and resulted in a parent response. As the child matured, the number of awakenings did not decrease significantly. However, more and more of the awakenings were briefer in length and were not associated with crying. Rather, the older infants were better able to fall back asleep on their own without a parental intervention. These observations led us to classify crying awakenings as “signaling awakenings” and quiet awakenings as “self-soothing awakenings”. The developmental shift from signaling to self-soothing awakenings began to appear around 2 months of age. The sleeping parent was unaware of these self-soothing awakenings and, thus, when these awakenings predominated, parents reported that their infant had slept through the night. Our data suggested, however, that even by 1 year of age, no infant truly slept through the night. We made two further observations from our video records. First, those infants who used a “sleep aid” (pacifier, thumb, soft cloth, etc.) while falling asleep were likely to reach for it when they awakened and use it to help them return to sleep. Second, infants who matured early as self-soothers around 3–4 months of age (the “good sleepers” according to their parents) often became signalers between 9 and 12 months of age. That is, as they approached their first birthday, the early good sleepers were likely to develop a night waking “sleep problem” [15, 19–21].

As we took note of the shifts in these middle-of-the-night signaling to self-soothing awakenings, we also began to take note of the sleep onset behaviors at the beginning of the night. Here, too, we noticed a developmental trajectory. During their early months, the large majority of infants were put into their cribs already asleep. Presumably, the infant had fallen asleep while feeding or being rocked and was then gently placed already asleep into the crib. Again, starting at around 2–3 months of age, we noticed that some infants were being put into their cribs still awake, perhaps drowsy, but still awake. These infants then fell asleep on their own. The transition from being put into the crib asleep to being put into the crib awake was gradual and non-linear. Sometimes the infant aroused after lying awake and required additional parental comforting outside of the crib. Sometimes parents did not remove their infant but stood next to the crib soothing their wakeful infant. The observation that surprised us was that the infants who were put into their cribs still awake and were able to fall asleep on their own, were the infants who were more likely to be able to put themselves back to sleep after a nighttime awakening. That is, they were significantly more likely to have more self-soothing awakenings after they had “learned” to fall asleep on their own at sleep onset. Or, in other words, infants who had “learned” to fall asleep at the beginning of the night were able to repeat this behavior in the middle of the night. They had “learned” how to self-soothe after a nighttime awakening. We also noted that infants who had learned to use a “sleep-aid” such as a fuzzy blanket, a pacifier and/or even their fingers or thumbs when falling asleep were more likely to use the same sleep aid in self-soothing returns to sleep later in the night.

**Figure 1.**
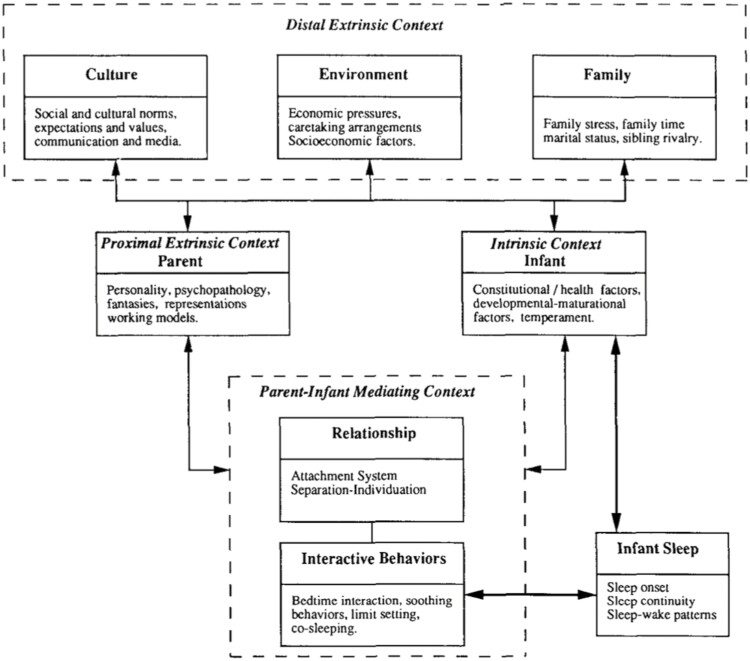
A transactional model: infant sleep from a systems perspective: proximal and distal effects on the development of sleep–wake patterns and sleep problems (from infant mental health journal, 14:17-34, 1993.).

These observations reawakened the work from my time at the Center for Advanced Studies in the Behavioral Sciences at Stanford, highlighting the importance of parent-infant interactions and contextual factors in early development. Serendipitously, from 1990 to 1992, as we were making these observations from our video recordings, Avi Sadeh, PhD was a visiting clinical research fellow in Mary Carskadon’s sleep lab, developing an algorithm for scoring infant sleep from actigraphic recording. Avi (whom we sadly lost when he was way too young) and I spent a great deal of time talking about the many contextual factors affecting infant sleep and posited a Transactional Model of Infant Sleep that thereafter guided both our research and clinical efforts (Figure 1).

## The Attachment Relationship and Infant Sleep

This was also the era in which much research had appeared on the importance of the parent-infant attachment relationship for healthy infant development. The nature of attachment security is assessed when the infant is around 1 year of age by what is known as the Ainsworth Strange Situation [18]. Essentially, mothers (fathers too) and their infants are observed during a sequence of separations and reunions in a structured laboratory setting. The infant’s responses to the stress of the mother’s leaving the room (separation) and the infant’s comfort response to her returning (reunion) and to the stress of a laboratory assistant’s presence (a stranger) determine the relationships’ dyadic attachment classification.

It occurred to me that each night an infant and her parent, from the earliest of ages, experience a separation and reunion sequence. Saying good night and separating each night may be stressful for both infant and parent and returning to a fussy infant is a reunion to provide comfort and security. In other words, a form of the Ainsworth Strange Situation, testing the attachment relationship, is relived each night and sometimes multiple times each night by the parent-infant dyad. The parent’s manner of, and response to, separating and reuniting each night might well be a factor in how the infant learns about signaling and self-soothing. Sadeh and his students and colleagues subsequently published many studies examining the effects of parental characteristics, such as interaction styles, cognitive styles, emotional availability and attachment types that affect infant sleep [22–29].

## Clinical Implications

Our video observations of sleep onset and night waking behavior and our Transactional Model greatly influenced our clinical work. When parents presented an infant with problematic sleep, older than four months of age, a careful developmental, interpersonal, cultural and contextual history (whenever possible from both parents), plus a 2-week sleep diary and a video recording when necessary guided our attempt to develop a personalized family treatment plan. The plan was offered only after a positive relationship had been fostered with the parents, a clear understanding of the parents’ concerns and prior responses had been obtained, and an educational introduction to the principles of sleep–wake development in infants had been provided. In general, we emphasized a gradual introduction of putting the infant into the crib awake at sleep onset; a 3–5-minute delay before responding to a signaled awakening; and, the use of a sleep aid in the crib. But we tailored these suggestions and the speed of their implementation to fit the needs, beliefs and capabilities of a particular family. For the very few intractable situations that we encountered, we sent a “somnotherapist,” usually an undergraduate student from our lab, to the home to support the family’s bedtime routine. In general, our personalized clinical interventions, provided to families of infants 4 months to 3 years of age, were well received and successful over the long term.

## The Transitional Object and Sleep

In 1992, my lab moved for the last time to University of California (UC) Davis where I became Chair of the Department of Psychiatry. In Davis, our group continued to focus on the problem that plagued so many families; namely, how to get babies to fall asleep on their own, to consolidate their sleep and to develop self-soothing awakenings. Research had previously reported that a newborn’s olfactory apparatus was functional at birth. Week-old newborns were able to recognize their mother’s pheromones as demonstrated by their turning their heads in the direction of a breast pad saturated with their mother’s breast milk in contrast to a pad saturated with a stranger’s breast milk [30]. Moreover, we had observed how some infants used their sleep aid (often a smelly object) while falling asleep and when returning to sleep after an awakening. The sleep aid seemed analogous to the “transitional” object, described by Bowlby and Winnicott to ease the stress of separations [17, 31]. Moreover, “blankies,” favorite teddy bears, etc. were well known treatments for fussy infants from prior generations. Presumably, the soothing qualities of these transitional objects are associated with their familiar odors since there is often much opposition on the part of the toddler to washing the object.

Incorporating this paradigm, we designed the longitudinal “smelly T-shirt” study. We enrolled a group of primiparous, breast feeding mothers and their newborn infants when the infant was one month of age. Mothers in the intervention group were given an extra-large T-shirt, marked with a black and white checkerboard pattern for easy video viewing. They were asked to wear this T-shirt as a night shirt for three consecutive nights while they slept and while they nursed their infant. We assumed that this T-shirt would be rich with maternal pheromones. The odoriferous T-shirt was then knotted into a small “ball” and placed in the infant’s crib. Our video camera recorded the infants’ sleep and their use of the T-shirt during the night to facilitate sleep onset and self-soothing awakenings. At the end of each month, from 1 month to 12 months of age, the mother washed the T-shirt, re-wore it for 3 nights and replaced the refreshed odoriferous T-shirt into the crib. Each mother had two T-shirts so she could remove, wash and replace one of them without depriving the infant of a T-shirt during the end-of-month transition. A matched “sham” control group of mothers and infants was given a T-shirt that was not worn by the mother, but rather merely knotted and placed into the crib. Each month a fresh T-shirt replaced the previous one. This sham T-shirt, however, also became impregnated with the infant’s own odors [32]. Unfortunately, the smelly T-shirt intervention was not significantly correlated with the development of more self-soothing awakenings and, thus, we were not able to discover a way to impose a transitional object on an infant [33–36]. As Bowlby had long ago posited, an infant must choose her own object [17].

## Sleep in Young Autistic Participants

In the final years before retirement, the MIND Institute, a research center dedicated to the study of autism and other neurodevelopmental disorders was founded at UC Davis. There, our group mounted a study on the sleep of young children with autism compared to children with neurodevelopment delay without autism and to typically developing children, all matched for age and gender. All of these children were recruited from the general population and were not particularly selected for having a sleep problem.

This study also utilized a new real time, hard drive video camera that could record an entire night’s sleep on a small computer chip embedded in the camera. The infra-red-light source also had been incorporated in the camera. So, no longer did we need to haul the cumbersome time-lapse video recorder and all the associated equipment and cords into the home. A compact camera on a tripod next to the crib was sufficient. In this study we also simultaneously recorded sleep with an actigraph attached to the child’s ankle so that we could compare actigraphic scoring algorithms and our manual video coding. Although, the mechanical aspects and logistics of real time video recording had become significantly easier and user friendly, the manual coding of a whole night of sleep (6–8 hours) in real time could still take 3–4 hours in the laboratory. In this study, we reported that the sleep of autistic preschoolers without a designated sleep problem did not differ significantly from the other two comparison groups in terms of sleep onset times and the number of nighttime awakenings [37].

Since retiring in 2007 and wrapping up the autism study at UC Davis in 2009, I became aware of an automated video coding system for infant sleep that is based on Sadeh’s actigraph algorithm. Assaf Glazer, an Israeli vision-computer scientist, worked with Sadeh to translate video pixels into movement epochs that could then be translated into automated video sleep–wake coding. A number of polygraphic and actigraphic studies have now validated the video coding algorithm [38–40]. Assaf founded a commercial start-up company, Nanit, which produces a high quality, compact video camera that is purchased by parents (https://www.nanit.com). The Nanit system records and codes sleep, night after night, longitudinally in developing infants and stores the raw data in the cloud. The algorithmically coded night time summaries, also in the cloud, are available to interested parents. In addition, many of the Nanit families participate in ongoing research projects. In full disclosure, I have become a member of Nanit’s Scientific and Medical Advisory Board and advise the research division, *Nanit Lab*, headed by Natalie Barnett PhD, on ongoing and future research opportunities. The hundreds of thousands of longitudinal nights in the cloud is truly a gold mine of developmental and epidemiologic data and the international Nanit user group is similarly a gold mine of potential research participant families with infants from birth to usually 3 years of age. So, in my retirement, I remain excited by the opportunities in infant sleep research that continue to unfold.

## Summary

It is difficult to assess one’s own legacy. I feel that I was lucky to be present at the very beginning of infant sleep research, learning from Howard Roffwarg, MD and Joseph Muzio, PhD. I was also present when pediatric sleep disorders, including the behavioral insomnias of early childhood, were being discovered. I hope that I have been able to contribute to both of these fields. A manual for polygraphically scoring infant sleep started me off, establishing home video recording and scoring of infant sleep–wake states followed. My focus on video recording has progressed from time-lapse video recording through hard drive, real time recording, to automated algorithmically coded video recording and cloud-based storage.

Using the earlier methods of video recording and manual scoring, we learned that infants and toddlers, like adults, don’t sleep uninterruptedly through the night but rather awaken multiple times. Nighttime awakenings gradually transform from signaled awakenings (crying) to self-soothing awakenings (a return to sleep without crying). With Avi Sadeh, we suggested a Transactional Model of sleep–wake development that highlights the importance of multiple cultural, contextual, interpersonal and individual factors associated with the emergence of consolidated sleep and optimal sleep habits. The Transactional Model has fostered many research studies and has contributed to personalized assessment and treatment of the early infant behavioral insomnias. It has been my good fortune to have been an early participant in, and contributor to, the current immense enterprise that today is Sleep Medicine.

## Notes

*This paper is part of the Living Legends in Sleep Research series, which is sponsored by Idorsia Pharmaceuticals and Jazz Pharmaceuticals.*
